# Two in One: Size Characterization and Accelerated Short-Term Physical Stability of Dual-Drug Suspensions with Two Acidic Compounds (Indomethacin and Naproxen)

**DOI:** 10.3390/pharmaceutics16121495

**Published:** 2024-11-21

**Authors:** Nadina Zulbeari, Signe Malig Hansen, René Holm

**Affiliations:** Department of Physics, Chemistry, and Pharmacy, University of Southern Denmark, Campusvej 55, 5230 Odense, Denmark; nazu@sdu.dk (N.Z.); siha521@student.sdu.dk (S.M.H.)

**Keywords:** long acting injectables, media milling, nano- and microsuspensions, dual centrifuge, dual compound, physical stability

## Abstract

**Background/Objectives:** Co-delivering dual-drug systems have proven to be effective in, for example, anticancer therapy or HIV prophylaxis due to a higher target selectivity and therapeutic efficacy from compound synergism. However, various challenges regarding physical stability can arise during the formulation definition when multiple drug compounds are included in the same formulation. In this work, the focus was on aqueous suspensions, which could be applied as long-acting injectable formulations to release the drug compounds over weeks to months after administration. **Methods:** It was possible to gain insights into dual-drug nano- and microsuspensions containing two acidic compounds (indomethacin and naproxen) prepared by milling with dual centrifugation. Information regarding the physical stability of individual suspensions was subtracted and compared to dual-drug suspensions when prepared with the same milling conditions and stored at elevated temperatures of 40 °C. **Results:** Distinct particle size profiles after milling were obtained dependent on the stabilizer used in both individual and dual-drug suspensions. Most notably, the combination of indomethacin and naproxen in one formulation resulted in smaller sizes of drug particles compared to individual suspensions under the presence of some stabilizers. The obtained particle size profiles further indicated that at least one of the model compounds needed to be sufficiently stabilized from a stabilizer to obtain physically stable dual-drug suspensions over 28 days when stored at 40 °C. Similarly, the particle size distribution was dependent on the individual distribution of the suspensions, which showed a monomodal distribution could be achieved for dual-drug suspensions when at least one of the individual suspensions demonstrated a monomodal distribution in the presence of the stabilizer alone. Over a 28-day period, the smallest particle size was obtained in dual-drug suspensions stabilized with a combination of polysorbate 85 and poloxamer 338 compared to dual-drug suspensions stabilized with only a single stabilizer during preparation, indicating tendencies towards stabilization synergism from a combination of stabilizers as well as the model compounds. **Conclusion:** Overall, the study showed insights into the preparation and physical stability of dual-drug suspensions containing indomethacin and naproxen.

## 1. Introduction

Treatment of mental conditions (e.g., schizophrenia) or infectious diseases (e.g., human immunodeficiency virus (HIV)) are particularly vulnerable to suboptimal patient compliance due to the risk of symptom recurrence, relapse probability and risk of hospitalization. The risk of poorer long-term clinical outcomes is thereby ultimately increased due to the frequent administration of a cocktail of drug compounds daily [1,2,3,4,5]. For that purpose, different formulation strategies have been thoroughly explored to reduce the dosing frequency in an attempt to improve non- or partial compliance and thereby enhance long-term clinical performance [4,6,7]. Here, long-acting injectables have been found to exhibit the effectual ability to maintain a safe and steady release of an active pharmaceutical compound for a prolonged period of time from a single injection administered subcutaneously or intramuscularly every month to a few months, thereby reducing a high dosing frequency [6,7,8,9,10,11,12].

One of the main platforms for the preparation of long-acting injectables is aqueous nano- and microsuspensions of crystalline drug particles. These suspensions are engineered to obtain a release rate for a desired plasma concentration profile. They are associated with several favorable attributes with their simple formulation design and manufacturing. Other advantages include a high drug load per injection, less excipient dependency and variability, as well as particle size control and optimization to achieve a desired in vivo performance [2,4,9,10,11,12,13].

Aqueous nano- and microsuspensions are generally manufactured by either bottom-up or top-down approaches and occasionally even a combination of both [4,14,15,16,17,18,19,20]. The use of top-down approaches, such as wet bead media milling, are typically employed and preferred due to the possibility of controlling the sizes of drug particles suspended in an aqueous dispersion medium by adjusting the milling conditions (e.g., milling speed and bead size) and formulation composition with various options for stabilizing agents (e.g., polymers and/or surfactants) [17,21,22]. However, the stabilization efficiency and therefore, the physical stability are highly dependent on the affinity between the stabilizing agents and the specific drug compound. Variations between the combination of stabilizing agents and the concentrations/ratios during preparation are, therefore, often observed depending on the drug compound used [17,23]. The screening of adequate stabilizing composition during the preparation of nano- and microsuspensions containing a single drug compound is, therefore, often time-consuming. Small-scale screening studies are typically preferred and conducted initially to evaluate the stabilization efficiency for formulation definition [24,25]. Similarly, screening studies for dual-drug formulations would likewise necessitate highly extensive screening studies where the affinity and interaction between one or several stabilizing agents and two drug compounds would need to be considered. However, the latter would be crucial for dual compound nanosuspensions, which could be favorable when the treatment plan contains a cocktail of several drug compounds. Co-delivering multiple pharmaceutical active compounds with different mechanisms in a single formulation [26] has gained more attention due to its clinical and operational benefits. In some cases, a higher therapeutic efficacy, target selectivity and reduced side effects could be obtained from compound synergism during, for instance, anticancer therapy, autoimmune diseases, or HIV prophylaxis [26,27,28,29,30,31,32,33]. A study by Youssef et al. [30] investigated the preparation of dual-drug-loaded nanostructured lipid carriers for the treatment of mixed ocular infections with ciprofloxacin and natamycin. Another study by Haloi and coworkers [31] tried to prepare an in situ hydrogel formulation containing methotrexate and phenethyl isothiocyanate for the treatment of rheumatoid arthritis. More recently, a study by Jin and colleagues [32] employed experimental and computational methodologies to design and synthesize dual-drug-loaded polymer nanoparticles. The study presented different challenges regarding co-encapsulation during the preparation of dual-loaded nanoparticles, where compounds with distinct hydrophobic properties exhibited challenges during co-encapsulation.

However, to the best of our knowledge, no studies in the literature explored the physical stress stability of dual-drug, aqueous nano- or microsuspensions prepared by dual centrifugation. For that reason, understanding the physical stability of individual suspensions containing a single drug compound was of interest to investigate and gain some insights into the considerations needed during the preparation of dual-drug suspensions. Suspensions were therefore prepared with two different chemically acidic model compounds, i.e., indomethacin or naproxen, to determine adequate stabilizer conditions of the individual model compounds and translated to suspensions containing both model compounds by investigating the short-term physical stability of dual compound suspensions. Thus, other formulation characteristics and considerations, such as the chemical stability of the prepared suspensions, were not covered in the present study.

## 2. Materials and Methods

### 2.1. Materials

Naproxen (purity: 98%) was obtained from abcr GmbH (Karlsruhe, Germany) and indomethacin (γ-form, purity: 99.8%) from Tokyo Chemical Industry Co., Ltd. (Tokyo, Japan). Acetic acid glacial (>97%) was purchased from Fisher Scientific (Loughborough, UK) and citric acid from VWR Chemicals (Leuven, Belgium). Polysorbate 20 (Thermo Scientific, Geel, Belgium), polysorbate 80 (Sigma-Aldrich, St. Louis, MO, USA), polysorbate 85 (Sigma-Aldrich, St. Louis, MO, USA), poloxamer 188 (Thermo Scientific, Kandel, Germany), poloxamer 338 (BASF, Ludwigshafen, Germany), poloxamer 407 (BASF, Ludwigshafen, Germany), sodium dodecyl sulfate (VWR Chemicals BDH, Leicestershire, UK), vitamin E D-α-tocopherol polyethylene glycol 1000 succinate (TPGS) (Sigma-Aldrich, St. Louis, MO, USA), polyvinylpyrrolidone (PVP) K16-18 (Thermo Scientific, Fair Lawn, NJ, USA), and PVP K30 (Thermo Scientific, Fair Lawn, NJ, USA), were purchased as stabilizers. The water quality was highly purified and obtained from an Ultrapure Water dispenser (18.2 MΩ, 25 °C, Merck, Millipore, Guyancourt, Yvelines, France). VitaBeads Nano (ø 1.0 mm, bulk density: 3.6 kg/L) were purchased from NETZSCH (yttrium-stabilized zirconium oxide beads, Selb, Germany) and used as milling beads. Acetonitrile was purchased from VWR Chemicals (Rosny-sous Bois-cedex, France).

### 2.2. Suspension Preparation by Dual Centrifugation

Stabilizer solutions were prepared with 3% (*w*/*v*) stabilizer dissolved in 50 mM acetate buffer, pH 4.0, or 50 mM citric buffer, pH 3.0. Suspensions were prepared in 2 mL micro twist tubes filled with 100 mg drug compound (indomethacin, naproxen, or a mixture of both), 1.0 g milling beads (ø 1.0 mm), and 1.0 mL stabilizer solution.

Milling was performed with a dual centrifuge (DeltaVita^®^ 1, NETZSCH, Selb, Germany). A single milling cycle was 90 min at 1500 rpm with a rotor temperature set to 0 °C. The milled suspensions were separated from the milling beads and stored in closed glass vials at 40 °C for 0, 7, 14, and 28 days while protected from light, followed by particle size measurements for the physical stability assessment.

### 2.3. Particle Size Measurements

Particle size profiles of prepared suspensions were measured after 0, 7, 14, and 28 days of storage at 40 °C with laser diffraction using a Malvern Mastersizer 3000 (software version 3.84. Malvern Instruments, Malvern, UK). The laser diffractometer was connected to a hydro medium-volume unit, working as a wet dispersion unit linked to water with a stirring rate set to 1200 rpm during measurements. The pH of the dispersant was lowered to pH 3 or pH 4 with glacial acetic acid during measurements to avoid solubilization of the drug compounds in water.

The optical parameters during measurements were adjusted depending on the drug compound. For suspensions prepared with only naproxen, the refractive index was set to 1.61 with a particle density of 1.20 g/cm^3^, whereas the refractive index was set to 1.68 with a particle density of 1.32 g/cm^3^ when suspensions were prepared with only indomethacin. The absorption index was set to 0.1 for both compounds, and the refractive index of water was set to 1.33. Each suspension was measured five times.

Particle size profiles with the corresponding *d*-values and span (Equation (1)) of the suspensions containing only indomethacin and naproxen were assessed and interpreted with a volume-based diameter approach based on Mie’s theory for non-spherical particles. Measurements were reanalyzed to determine if air bubbles interfered with the obtained particle size profiles [34].

Span = (D_90_ − D_10_)/D_50_ (1)

#### Analysis of Particle Size Profiles of Dual-Drug Suspensions

To correctly analyze dual compound suspensions containing naproxen and indomethacin, the particle size profiles were both analyzed with the volume-based diameter approach based on the Mie theory of non-spherical particles with a refractive index set to 1.645 (average value of both compounds) and a particle density of 1.26 g/cm^3^ (average value of both compounds). The Opaque Particle Approximation (Fraunhofer Approximation) was also estimated without taking the optical parameters into account.

### 2.4. Thermodynamic Solubility in Dispersion Media

The thermodynamic solubility of naproxen and indomethacin was determined by reversed-phase, ultra-violet high-performance liquid chromatography (UV-HPLC) with isocratic elution (Agilent 1100 series, Agilent, Santa Clara, CA, USA) and a Waters XBridge BEH C18 column (130 Å, 3.5 µm, 4.6 mm, 150 mm). The mobile phase consisted of 50% acetonitrile and 50% purified water with a flow rate set to 1 mL/min and a column temperature set to 30 °C. The injection volume was 20 µL analyzed at a wavelength set to 230 nm for all analytical measurements.

Samples were prepared by the shake-flask method in glass vials containing 2 mg drug compound and 1 mL dispersion medium consisting of either buffer solutions (50 mM acetate buffer, pH 4.0 or 50 mM citric buffer, pH 3.0) or stabilizer solutions containing 1%, 2%, or 3% polysorbate 20 or vitamin E TPGS dissolved in the buffer solutions. The samples were rotated for 24 h at 40 rpm on a Multi-Rotator (PTR-60, Grant-Bio, Grant Instruments, Cambridgeshire, UK) while stored at 40 °C and protected from light. Samples were prepared in triplicate (n = 3). After equilibration, the samples were filtered with a 0.22 µm hydrophobic polytetrafluoroethylene membrane (VWR International, LLC, Radnor, PA, USA) with sampling tools equilibrated to 40 °C while the clear supernatant was diluted to an appropriate concentration with the mobile phase (50:50 acetonitrile and purified water) and analyzed by the method described above.

## 3. Results and Discussion

### 3.1. Preliminary Solubility Investigation

Indomethacin and naproxen are both weak acids with pKa-values of 4.5 and 4.2, respectively. The solubility of naproxen is highly pH-dependent on the surrounding environment due to ionization of the carboxylic group at higher pH, resulting in increased solubility, which normally would be favorable in oral drug delivery [35,36]. However, during the preparation of nano- and microsuspensions used as a platform for the preparation of long-acting injectables, the free base of the crystalline drug compound is typically preferred as this often has the lowest solubility and chance of disproportionation. Therefore, preliminary solubility experiments were performed to determine the solubility of both model compounds in different stabilizer vehicles dissolved in two different buffer systems, i.e., 50 mM citric buffer (pH 3.0) or 50 mM acetate buffer (pH 4.0). The most optimal buffer conditions to maintain uncharged groups during suspension preparation were of interest to identify. Additionally, the solubility of indomethacin and naproxen was investigated with increasing polysorbate 20 and vitamin E TPGS concentrations dissolved in either citric buffer, pH 3.0 or acetate buffer, pH 4.0, to determine if the solubility of the drug compounds would increase under the presence of different stabilizers due to the potential impact on the short-term physical stability during storage.

**Figure 1:**
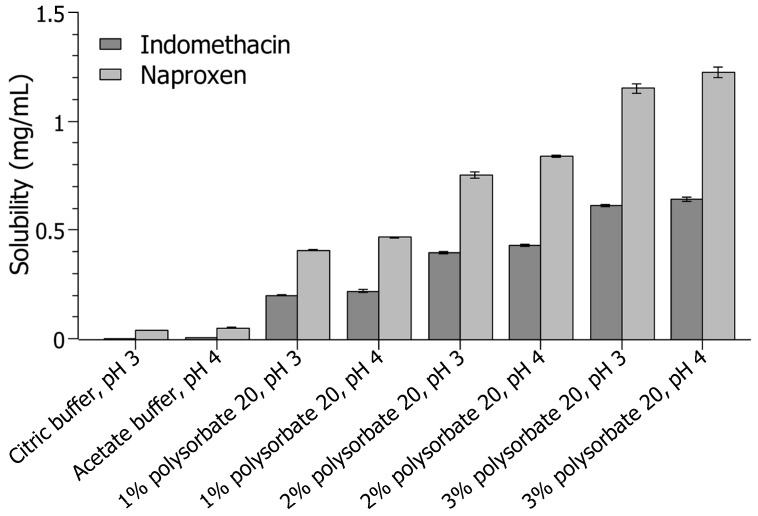
Overview of the solubility of indomethacin and naproxen with increasing concentrations of polysorbate 20 dissolved in either 50 mM citric buffer, pH 3.0 or 50 mM acetate buffer, pH 4.0 (n = 3).

In general, the obtained results demonstrated that the solubilities of both model compounds were higher when prepared with dispersion vehicles dissolved at higher pH, i.e., acetate buffer, pH 4.0, compared to the dispersion media at lower pH (Figure 1). The solubility of indomethacin was very low in the presence of both buffers, with a solubility of 0.002 mg/mL and 0.003 mg/mL in citric buffer, pH 3.0, or acetate buffer, pH 4.0, respectively. For naproxen, the solubility in the buffer systems was higher than for indomethacin, with a solubility of 0.037 mg/mL and 0.049 mg/mL in citric buffer, pH 3.0, or acetate buffer, pH 4.0, respectively. For that reason, stabilizer solutions used to prepare suspensions in the present study were dissolved in citric buffer with pH 3.0 to minimize solubilization of the drug compounds during nanomilling.

The same trend with increased drug solubility at higher pH was also observed during the presence of polysorbate 20. The drug solubility was higher in all cases when the stabilizer vehicle was dissolved at pH 4.0 rather than pH 3.0. Similarly, the solubility of both drug compounds was also higher in the presence of vitamin E TPGS with increased concentration, see the Supplementary Materials (see Figure S1). It was further observed that the solubility of naproxen was especially influenced by the dispersion medium and the presence of polysorbate 20. The highest concentration of polysorbate 20, i.e., 3% (*w*/*v*), led to a solubility above 1.20 mg/mL for naproxen, which was distinctly higher than the solubility of indomethacin. There, the highest solubility was determined to be 0.64 mg/mL at 3% (*w*/*v*) polysorbate 20 dissolved in acetic buffer with pH 4.0 (Figure 1). Similar solubility results for indomethacin suspensions stabilized with polysorbate 20 were obtained in a study by Zulbeari and colleagues [37], i.e., approximately 0.70 mg/mL with 4% (*w*/*v*) polysorbate 20, whereas the solubility of indomethacin was determined to be much lower in the presence of poloxamer 188, i.e., 0.50 µg/mL. The obtained results in the present study thereby indicated that the polysorbates and vitamin E TPGS greatly influenced the solubility of the model compounds, which ultimately could alter the physical stability during storage at elevated temperatures.

### 3.2. Screening of Stabilizers

It has recently been reported in the literature that different model compounds with significantly distinct hydrophobic properties can pose challenges during co-encapsulation in nanoparticles when aiming to produce dual-drug nanoparticles [32]. To gain an understanding of the physical stability of dual-drug nano- and microsuspensions, individual suspensions containing only indomethacin or naproxen were, for that reason, initially produced to support the stabilizer compositions during the preparation of dual-drug suspensions. Therefore, 10 different types of stabilizers comprised of both surfactants and polymers, i.e., polysorbate 20, polysorbate 80, polysorbate 85, poloxamer 188, poloxamer 338, poloxamer 407, PVP K16-18, PVP K30, vitamin E TPGS, and SLS, were initially screened for the stabilization of indomethacin or naproxen suspensions. The obtained data was subsequently compared to dual-drug suspensions containing both model compounds. The milling conditions were kept constant (90 min, 1500 rpm, 0 °C cooling temperature) with 1.0 g milling beads (ø 1.0 mm) as well as the formulation parameters (100 mg drug compound, 1 mL stabilizer solution of 3% (*w*/*v*)) throughout the present study.

**Figure 2:**
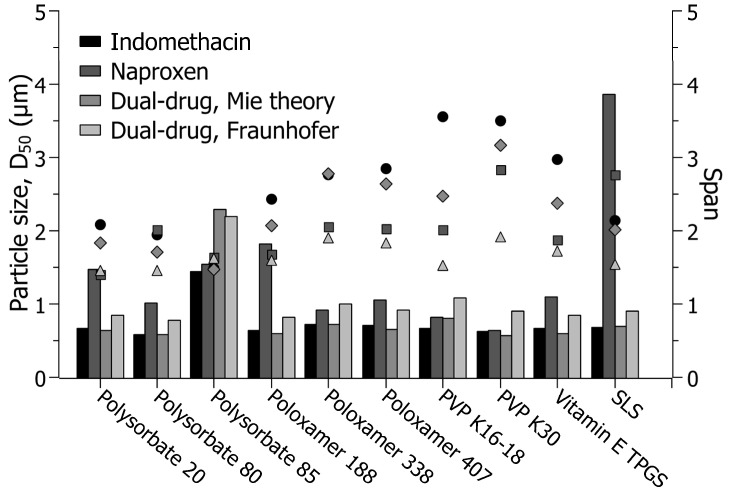
Overview of obtained sizes of particles (D_50_-values) (µm) from the stabilizer screening (dissolved in 50 mM citric buffer, pH 3.0) of prepared suspensions containing indomethacin, naproxen, or dual-drug suspensions of both compounds analyzed with either Mie’s theory or the Fraunhofer approximation. The span values are presented as symbols with indomethacin as the ellipse symbol, naproxen as the rectangular symbol, dual-drug suspensions analyzed with Mie’s theory as the diamond symbol, and dual-drug suspensions analyzed with the Fraunhofer approximation as the triangular symbol.

It was possible to decrease the sizes of drug particles of suspensions prepared in the presence of all 10 stabilizers used during nanomilling by dual centrifugation compared to unmilled drug particles. An overview of the sizes of unmilled drug particles for comparison can be found in the Supplementary Materials (Table S1). Thus, the particle size profiles of indomethacin or naproxen after milling of the individual suspensions were highly dependent on the stabilizer used during preparation. However, it was possible to draw some general correlations between the prepared suspensions. Smaller sizes of indomethacin particles were generally achieved in the presence of all 10 stabilizers as compared to naproxen suspensions (Figure 2). Polysorbate 85 showed poor stabilization properties immediately after milling for both drug compounds, whereas the remaining stabilizers showed similar stabilization properties for indomethacin suspensions immediately after milling since similar sizes of drug particles were observed for the remaining stabilizers (Figure 2). Similar particle size distributions of indomethacin suspensions were also achieved for all stabilizers except for polysorbate 85, correlating to the obtained sizes of indomethacin particles, which can be found in the Supplementary Materials (Figure S2).

As for naproxen suspensions, polysorbate 20 and polysorbate 85 did not seem to stabilize particles sufficiently during nanomilling compared to the other stabilizers. These observations could be explained by larger sizes of particles obtained, i.e., D_50_-values on 1.47 µm and 1.54 µm with polysorbate 20 and polysorbate 85, respectively (Figure 2). Poloxamer 188 and SLS similarly showed poor stabilization properties for naproxen suspensions with D_50_-values of approximately 2 or 4 µm for poloxamer 188 and SLS, respectively. However, it was interesting to note that dual suspensions prepared containing both indomethacin and naproxen, stabilized with either poloxamer 188 or SLS, produced significantly smaller particle sizes in the dual-drug suspensions (below 1 µm), indicating a stabilization synergism between indomethacin and naproxen or even modification of surface properties when both drug compounds were present during milling.

**Figure 3:**
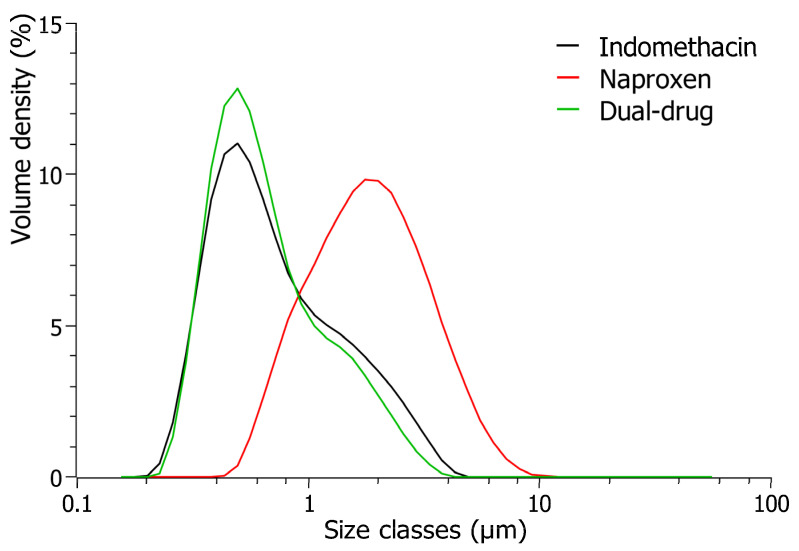
Particle size distribution of prepared suspensions containing either indomethacin (black), naproxen (red), or both compounds (green) stabilized with 3% (*w*/*v*) poloxamer 188 (dissolved in 50 mM citric buffer, pH 3.0). Particle size distributions were analyzed using Mie’s theory.

The observed trend was further supported by the sizes of drug particles obtained in dual suspensions analyzed with Mie’s theory. The combination of both drug compounds resulted in smaller sizes of drug particles when individual suspensions were prepared, as seen for the particle size distributions illustrated in Figure 3 for suspensions stabilized with poloxamer 188. The latter trend was additionally observed with polysorbate 20, polysorbate 80, poloxamer 407, PVP K30, and vitamin E TPGS, where smaller sizes of drug compounds were achieved in the dual-drug suspensions instead of the individual systems. Only dual-drug suspensions stabilized with polysorbate 85 increased by approximately 0.5 µm compared to the individual systems (Figure 2), which correlated to the poor stabilization properties observed for both indomethacin and naproxen suspensions when prepared individually. An overview of the remaining d-values immediately after milling for all prepared suspensions can be found in the Supplementary Materials (Tables S2–S31). The findings, therefore, suggest that poor stabilization was maintained in dual-drug suspensions directly after milling when both individual compounds had poor affinity towards the particular stabilizer. On the contrary, sufficient stabilization and decreased sizes of drug particles could be obtained in dual-drug suspensions directly after milling, as long as positive stabilization properties of the stabilizers were obtained for at least one of the model compounds during preparation, i.e., with the limited systems investigated, it appears that the affinity of the stabilizer towards just one of the compounds was sufficient to produce a smaller particle size of the combined suspension.

### 3.3. Interpretation of Dual-Drug Suspensions

To ensure proper analysis of particle size profiles of suspensions containing both model compounds, dual-drug suspensions were analyzed with both the Mie theory assessed from the average value of both compounds’ refractive indexes, i.e., 1.645, and estimated with the Fraunhofer approximation, which estimated the sizes of drug particles without considering the optical properties and thereby the refractive index.

**Figure 4:**
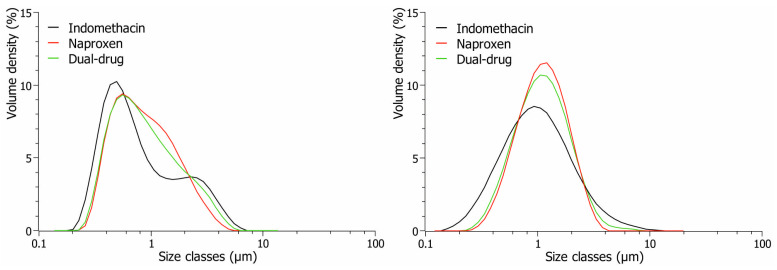
Particle size distribution of prepared suspensions containing either indomethacin (black), naproxen (red), or both compounds (green) stabilized with 3% (*w*/*v*) PVP K16-18 (dissolved in 50 mM citric buffer, pH 3.0). Particle size profiles were analyzed using Mie’s theory (**left**) or the Fraunhofer approximation (**right**).

When particle size profiles were estimated with the Fraunhofer approximation, suspensions prepared with all 10 stabilizers increased by approximately 0.2 µm in size compared to particle sizes obtained when suspensions were analyzed with the Mie theory, as seen previously in Figure 2. The difference in particle sizes correlated to the consideration of the compound’s optical parameters used in the Mie theory for more accurate size analysis. Not only did the obtained sizes of particles differ when analyzed with the two models, but different particle size distributions were further obtained depending on the models. Specifically, the particle size distribution of indomethacin varied depending on the model used for the analysis of the particle size profiles. When analyzed with the Mie theory, a monomodal distribution was observed right after milling for naproxen suspensions, while a somehow bimodal distribution with a coarse tail was observed for indomethacin suspensions stabilized with PVP K16-18 visualized in Figure 4 (left). The Fraunhofer approximation was additionally used for particle size analysis to exclude the optical parameter dependency and thereby estimate the sizes of drug particles when two different compounds were investigated with different optical parameters. However, during size analysis with the Fraunhofer approximation, the bimodal distribution of indomethacin suspensions was substituted with a monomodal distribution with smaller sizes of indomethacin present (Figure 4, black), indicating differences in the two models for size analysis. Similar trends were observed with the remaining stabilizers, except for polysorbate 85, where a fine tail toward lower size classes was observed for suspensions containing both drug compounds. Even with a more monomodal distribution for all suspensions when analyzed with Fraunhofer approximation, the model had its limitations during size analysis based on the sizes of milled drug particles. During analysis with the Fraunhofer approximation, the software on the Mastersizer 3000 indicated poor data quality and that the model was not well-suited for particle sizes below 2 µm. This was presumably explained by the small sizes of measured particles, i.e., <1 µm, which some of the milled suspensions had obtained after nanomilling since the model is often used for optically larger particles when the wavelength of the illuminating light is significantly smaller than the measured particle sizes [38,39]. Since the Mastersizer 3000 is equipped with a blue light source with a wavelength of 470 nm, the milled nanosuspensions were out of the detection range for the Fraunhofer approximation for accurate size approximation. For that reason, the particle size profiles of dual-drug suspensions were primarily analyzed using Mie’s theory and the average of the two compounds’ refractive indexes. However, the sizes of drug particles in dual-drug suspensions analyzed with Fraunhofer approximation can be found in the Supplementary Materials in Tables S22–S31.

As previously stated, differences in particle size distributions were observed when the prepared suspensions were analyzed using Mie’s theory, which was dependent on the stabilizer vehicle. A monomodal distribution was achieved for indomethacin suspensions when stabilized with polysorbate 20, polysorbate 80, polysorbate 85, poloxamer 188, vitamin E TPGS, and SLS (Table 1, green). A more bimodal distribution was observed for indomethacin suspensions when stabilized with poloxamer 338, poloxamer 407, and PVP K30 (Table 1, red). As for naproxen suspensions, only polysorbate 85, PVP K30, and SLS resulted in bimodal distributions (Table 1, red). The remaining stabilizers resulted in monomodal distributions for naproxen suspensions. Interestingly, when a monomodal distribution was achieved for both drug compounds, a monomodal distribution was also achieved for the dual-drug suspensions. This was the case for polysorbate 20, polysorbate 80, poloxamer 188, and vitamin E TPGS (Table 1). On the other hand, when a bimodal distribution was achieved for both individual suspensions, i.e., PVP K30, a bimodal distribution was also obtained for the dual-drug suspensions (Table 1). The obtained results thereby indicated that the distribution was dependent on the individual suspensions also, either resulting in a mono- or bimodal distribution dependent if at least one of the individual suspensions achieved a monomodal distribution, as seen for polysorbate 85, poloxamer 338, poloxamer 407, PVP K16-18, and SLS.

### 3.4. Short-Term Physical Stability Assessment

Sufficient stabilization of nano- and microsuspensions directly after milling needs to be predictable and comparable to the physical stability long-term [14]. The prepared suspensions were therefore subjected to stress conditions at an elevated storage temperature of 40 °C in closed glass containers with Teflon-lined caps to evaluate the short-term physical stability of the prepared suspensions when exposed to stress conditions to accelerate the physical stability assessment as per ICH guideline Q1 A (R2).

In all cases, the sizes of drug particles increased over the duration of 28 days of storage at elevated temperatures for suspensions containing only indomethacin or naproxen, and the dual-drug suspensions stabilized with all 10 stabilizers. Thus, the degree to which the particle sizes increased depends on the stabilizer vehicle used during preparation. Immediately after milling, the initial sizes of indomethacin particles ranged from approximately 0.590 µm to 0.720 µm, where only indomethacin particles stabilized with polysorbate 85 led to a particle size of 1.440 µm (Table 2). However, after 28 days of storage, only polysorbate 20, polysorbate 85, and PVP K16-18 led to particle sizes above 1 µm, whereas the remaining sizes of suspensions remained below 1 µm. As for naproxen suspensions, only suspensions stabilized with poloxamer 338, PVP K16-18, and PVP K30 led to particle sizes below 1 µm initially right after milling. Thus, all 10 suspensions increased to particle sizes above 1 µm after 28 days of storage (Table 2). An overview of the physical stability and the corresponding span and d-values for the remaining days (7 and 14 days) of the individual suspensions containing indomethacin or naproxen can be found in the Supplementary Materials (Tables S2–S21). In particular, polysorbate 20 resulted in larger sizes of drug particles. An increase from 0.662 µm to 1.230 µm and 1.470 µm to 3.950 µm for indomethacin and naproxen suspensions, respectively, was observed and from 0.632 µm to 1.720 µm for dual-drug suspensions (Table 2). The increase was the largest difference observed over time for the investigated dual-drug suspensions, together with the dual suspension stabilized with SLS. The increased sizes of drug particles were presumably due to the increased solubility of the drug compounds in the presence of polysorbate 20, as explained prior in Section 3.1. Ultimately, the increased solubility would lead to crystal growth and increased sizes of particles when stored at elevated temperatures, i.e., Ostwald ripening [40]. The obtained results further supported that naproxen suspensions were generally more unstable with a bigger increase in the sizes of particles, as also seen with the higher solubility in the presence of stabilizers and a larger increase in particle sizes over time. However, it must be noted that the largest increase in particle sizes of indomethacin and dual-drug suspensions occurred during the first week of storage at elevated temperature, i.e., 40 °C, since the sizes of particles were stable after one week of storage. The findings were most likely due to the stabilization equilibrium adjustment towards the elevated storage temperature, with a similar trend that has been reported previously in the literature by Zulbeari and coworkers [36,41]. On the contrary, the obtained results showed that the sizes of naproxen particles increased slightly throughout 28 days, indicating naproxen suspensions were more prone to instability (e.g., crystal growth by Ostwald ripening due to increased solubility) during storage, also supported by the generally larger particles observed over 28 days of storage compared to the remaining suspensions.

When dual-drug suspensions were prepared, the obtained short-term physical stability results indicated a synergistic stabilization effect in the presence of indomethacin and naproxen, with a modified affinity between drug particles when both compounds were present with a stabilizer. For example, the presence of indomethacin in the dual-drug suspensions appeared to enhance the physical stability of naproxen, as the particle sizes increased to a lesser degree in comparison to when naproxen suspensions were prepared individually. However, during storage, the overall increase in drug particle sizes was slightly higher in dual-drug suspensions when compared to indomethacin suspensions alone. Considering the instability of naproxen and its presence in the dual-drug suspensions, the observed increases in drug particle sizes in the dual-drug suspensions were much lower over 28 days, indicating more physically stable dual-drug suspensions than naproxen suspensions alone. Similar to pure indomethacin suspensions, only dual-drug suspensions stabilized with polysorbate 85 resulted in initial particle sizes above 1 µm directly after milling. Thus, the increase over 28 days was much lower when compared to the individual suspensions (Table 2), again supporting the improved stability of naproxen in the dual-drug suspensions.

Based on the stabilization efficiency observed from the prepared suspensions, dual-drug suspensions were also prepared with various combinations of different stabilizers. The purpose of this was to investigate if a synergistic stabilization effect was observed in the short-term physical stability of dual-drug suspensions, similar to how the presence of indomethacin improved the physical stability of naproxen during storage. Five different stabilizer combinations were therefore investigated by combining both stabilizers, which showed sufficient stabilization properties for the individual systems and the dual-drug suspensions. This also included stabilizer combinations containing polysorbate 85, for example, which generally showed a poorer stabilization efficiency for both compounds throughout the whole study.

**Figure 5:**
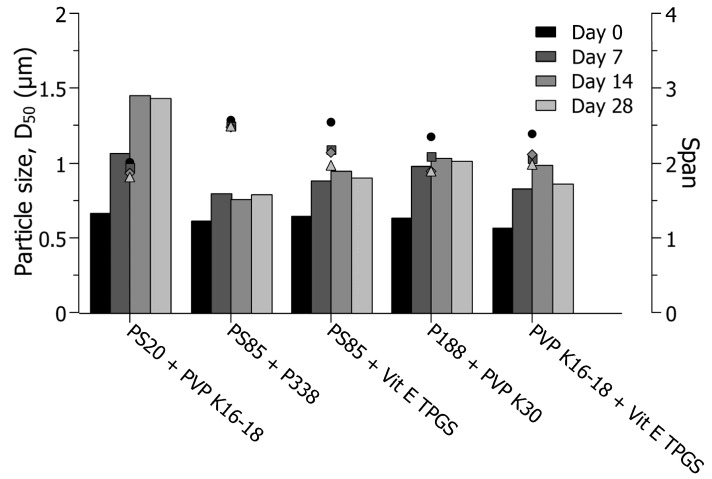
Overview of the short-term physical stability of dual-drug suspensions presented as particle sizes (d_50_-value, µm) over 28 days of storage at 40 °C. Suspensions were stabilized with 3% (*w*/*v*) (dissolved in 50 mM citric buffer) of either polysorbate 20/PVP K16-18 (PS20 + PVP K16-18), polysorbate 85/poloxamer 338 (PS85 + P338), polysorbate 85/vitamin E TPGS (PS85 + Vit E TPGS), poloxamer 188/PVP K30 (P188 + PVP K30), or PVP K16-18/vitamin E TPGS (PVP K16-18 + vit E TPGS). The span values are presented as symbols with day 0 as the ellipse symbol, day 7 as the rectangular symbol, day 14 as the diamond symbol, and day 28 as the triangular symbol.

As described above, the largest increase in drug particle sizes occurred during the first week of storage, followed by stable particle size profiles for the remaining weeks. Only dual-drug suspensions stabilized with a combination of polysorbate 20 and PVP K16-18 increased further between day 7 and day 14 (Figure 5), which was potentially caused by the presence of polysorbate 20 and the increased solubility of the drug compounds associated with polysorbate 20.

Throughout the present study, polysorbate 85 generally showed poor stabilization properties with limitations in size reduction and large increases in the sizes of drug particles. However, when polysorbate 85 was combined with poloxamer 338, the smallest increase in particle sizes occurred compared to the remaining combinations, with the final particle sizes being approximately 0.780 µm after 28 days of storage at 40 °C. The obtained particle size was smaller than the final particle sizes of dual-drug suspensions stabilized with either polysorbate 85, poloxamer 338 alone, or the remaining stabilizers individually. This observation indicated a stabilization synergism between polysorbate 85 and poloxamer 338, potentially explained by the strong interaction and structural changes in the mixed systems between the non-ionic surfactant (i.e., polysorbate 85) and the amphiphilic triblock copolymer (i.e., poloxamer 338) [42]. By combining polysorbate 85 and poloxamer 338, polysorbate 85 would adsorb in the interface between the core and shell of the formed micelles of poloxamer 338, which thereby could work as a spacer between the hydrophobic and hydrophilic chains of poloxamer 338, ultimately leading to a more thermodynamically stable system and forming a physically stable formulation [42,43]. As for the remaining combinations of stabilizers (Figure 5), i.e., polysorbate 20/PVP K16-18, polysorbate 85/vitamin E TPGS, poloxamer 188/PVP K30, and PVP K16-18/vitamin E TPGS, slightly larger particle sizes were observed compared to when dual-drug suspensions were stabilized with the respective stabilizers alone. The obtained results thereby demonstrated a positive effect of combining specific stabilizers during dual-drug suspension preparation containing indomethacin and naproxen.

The obtained results demonstrated new insights into the preparation of dual-drug suspensions using naproxen and indomethacin as model compounds. With a preliminary understanding of dual-drug suspensions and which factors need to be considered during formulation investigation, the present work showed promising indications that a system synergism could be obtained when at least one of the drug compounds had a positive stabilization efficiency. The presented work contributes to a baseline understanding of the physical stability of dual-drug suspensions by subtracting information from individual suspensions. Future studies are, thus, required to further expand the general understanding of dual-drug nano- and microsuspensions, which altogether could aim to improve the potential of these systems containing more than one drug compound in one suspension.

## 4. Conclusions

This study showed promising insights into the preparation of dual-drug nano- and microsuspensions containing indomethacin and naproxen as model compounds. It was possible to subtract information regarding the physical stability of the individual systems and project them into consideration during dual-drug suspension preparation. The increased solubility of indomethacin and naproxen at pH 4.0 compared to pH 3.0 was confirmed, and solubility was increased in the presence of polysorbate 20 and vitamin E TPGS. When prepared under similar milling conditions and stabilizer concentrations, distinct particle size profiles of indomethacin, naproxen, or dual-drug suspensions were obtained dependent on the stabilizer used. Smaller drug particle sizes were achieved immediately after milling (i.e., day 0) when dual-drug suspensions were stabilized with polysorbate 20, polysorbate 80, poloxamer 188, poloxamer 407, PVP K30, and vitamin E TPGS in comparison to suspensions containing either indomethacin or naproxen alone. The results further indicate that at least one of the drug compounds needs positive stabilization efficiency from a stabilizer to obtain physically stable dual-drug suspensions. Similar tendencies were further supported by the particle size distributions, which indicate a monomodal distribution was achieved during dual-drug preparation when a monomodal distribution was achieved for the individual suspensions. Correspondingly, a bimodal distribution of the individual suspensions led to a bimodal distribution of dual-drug suspension, while at least a monomodal distribution of one of the individual suspensions was necessary to achieve a monomodal distribution of dual-drug suspension. Polysorbate 85 exhibits tendencies toward poor stabilization efficiency of individual suspensions as well as dual-drug suspensions, explained by larger particle sizes and increased sizes over 28 days. Thus, positive stabilization efficiency was observed with polysorbate 85 when combined with poloxamer 338 in dual-drug formulations, where the smallest final particle size was obtained after 28 days of storage at an elevated temperature, indicating a synergistic stabilization effect between both the stabilizers and the two model compounds.

## Figures and Tables

**Table 1:** Color-coded overview of distributions of prepared suspensions directly after milling containing either indomethacin, naproxen, or both compounds stabilized with 10 different stabilizers. Green indicated a monomodal distribution, whereas red indicated a bimodal distribution. Suspensions were analyzed with the Mie theory.

	Indomethacin		Naproxen		Dual-Drug	
Stabilizer	Monomodal	Bimodal	Monomodal	Bimodal	Monomodal	Bimodal
Polysorbate 20						
Polysorbate 80						
Polysorbate 85						
Poloxamer 188						
Poloxamer 338						
Poloxamer 407						
PVP K16-18						
PVP K30						
Vitamin E TPGS						
SLS						

**Table 2:** Comparison of the particle sizes (d_50_-values, µm) of indomethacin, naproxen, or dual-drug (mix) suspensions with 3% (*w*/*v*) stabilizer (dissolved in 50 mM citric buffer, pH 3.0) from the screening of 10 different stabilizers right after milling and after 28 days of storage under stress conditions at elevated storage temperature, i.e., (40 °C).

	Indomethacin		Naproxen		Dual-Drug	
	Day 0	Day 28	Day 0	Day 28	Day 0	Day 28
**Polysorbate 20**	0.662 µm	1.230 µm	1.470 µm	3.950 µm	0.632 µm	1.720 µm
**Polysorbate 80**	0.590 µm	0.912 µm	1.020 µm	2.400 µm	0.578 µm	0.910 µm
**Polysorbate 85**	1.440 µm	2.620 µm	1.540 µm	4.880 µm	2.290 µm	2.950 µm
**Poloxamer 188**	0.642 µm	0.739 µm	1.820 µm	3.230 µm	0.596 µm	0.836 µm
**Poloxamer 338**	0.722 µm	0.857 µm	0.919 µm	2.460 µm	0.722 µm	0.927 µm
**Poloxamer 407**	0.705 µm	0.778 µm	1.060 µm	3.100 µm	0.650 µm	0.843 µm
**PVP K16-18**	0.665 µm	1.220 µm	0.816 µm	2.020 µm	0.803 µm	1.170 µm
**PVP K30**	0.619 µm	0.692 µm	0.637 µm	1.880 µm	0.565 µm	0.784 µm
**Vitamin E TPGS**	0.672 µm	0.757 µm	1.100 µm	1.790 µm	0.592 µm	0.878 µm
**SLS**	0.674 µm	0.858 µm	3.860 µm	4.120 µm	0.695 µm	1.870 µm

## Data Availability

The original contributions presented in the study are included in the article/Supplementary Materials; further inquiries can be directed to the corresponding author.

## Supplementary Materials

The following supporting information can be downloaded at: https://www.mdpi.com/article/10.3390/pharmaceutics16121495/s1, Table S1: Overview of sizes of unmilled drug particles. Tables S2–S11: Particle sizes over 28 days of prepared indomethacin suspensions stabilized with different stabilizers dissolved in citric or acetate buffer. Tables S12–S21: Particle sizes over 28 days of prepared naproxen suspensions stabilized with different stabilizers dissolved in citric or acetate buffer. Tables S22–S31: Particle sizes over 28 days of dual-drug suspensions stabilized with different stabilizers dissolved in citric buffer and analyzed with Mie’s theory or the Fraunhofer approximation. Table S32: Particle sizes of dual-drug suspensions stabilized with 3% (*w*/*v*) vitamin E TPGS and polysorbate 85 dissolved in citric buffer over 28 days of storage. Table S33: Particle sizes of dual-drug suspensions stabilized with 3% (*w*/*v*) poloxamer 188 and PVP K30 dissolved in citric buffer over 28 days of storage. Table S34: Particle sizes of dual-drug suspensions stabilized with 3% (*w*/*v*) poloxamer 338 and polysorbate 85 dissolved in citric buffer over 28 days of storage. Table S35: Particle sizes of dual-drug suspensions stabilized with 3% (*w*/*v*) vitamin E TPGS and PVP K16-18 dissolved in citric buffer over 28 days of storage. Table S36: Particle sizes of dual-drug suspensions stabilized with 3% (*w*/*v*) polysorbate 20 and PVP K16-18 dissolved in citric buffer over 28 days of storage. Figure S1: Solubility of indomethacin and naproxen in different buffer systems and increasing concentration of vitamin E TPGS. Figure S2: Particle size distribution of indomethacin, naproxen, or dual-drug suspensions right after milling.
