# Supplementary material for: Two in One: Size Characterization and Accelerated Short-Term Physical Stability of Dual-Drug Suspensions with Two Acidic Compounds (Indomethacin and Naproxen)
Source: Pharmaceutics. 2024 Nov 21;16(12):1495. doi: 10.3390/pharmaceutics16121495 (PMC11676886; doi:10.3390/pharmaceutics16121495)
Supplement: Supplementary file 1 [file pharmaceutics-16-01495-s001.zip › pharmaceutics-3267288-supplementary.pdf]

Two-in-One: Size Characterization and Accelerated Short-Term Physical Stability of Dual-Drug Suspensions with Two Acidic Compounds (Indomethacin and Naproxen)

Nadina Zulbeari[1], Signe Malig Hansen[1], René Holm[1*]

[1]Department of Physics, Chemistry, and Pharmacy, University of Southern Denmark, Campusvej 55, 5230 Odense, Denmark

*Corresponding Author:

René Holm, Department of Physics, Chemistry, and Pharmacy, University of Southern Denmark, Campusvej 55, 5230 Odense, Denmark. E-mail: reho@sdu.dk; Tel.: +45 9350 7033; Fax: +45 6550 1090

SUPPLEMENTARY MATERIALS

Tables

Table S1. Sizes of unmilled drug particles of prepared suspensions. Dual suspensions containing both indomethacin and naproxen were analyzed with both the Mie-Theory (MT) and Fraunhofer Approximation (FA).

	D_{10}	D_{50}	D_{90}	Span
**Indomethacin**	15.60	46.40	169.1	1.731
**Naproxen**	13.60	32.60	63.20	1.523
**Mix, MT**	13.40	36.50	98.00	2.311
**Mix, FA**	12.50	36.10	96.00	2.307

Table S2. Particle sizes of prepared indomethacin suspension stabilized with 3% (m/v) polysorbate 20 dissolved in either citric buffer, pH 3.0 or acetate buffer, pH 4.0. Suspensions were stored over 28 days at 40 °C.

	**Citric buffer, pH 3.0**				**Acetate buffer, pH 4.0**			
	**Day 0**	**Day 7**	**Day 14**	**Day 28**	**Day 0**	**Day 7**	**Day 14**	**Day 28**
$\mathbf{D_{10}}$	0.362	0.510	0.465	0.625	0.321	0.377	0.440	0.500
$\mathbf{D_{50}}$	0.662	1.140	1.020	1.230	0.531	0.758	0.957	1.090
$\mathbf{D_{90}}$	1.740	2.580	2.340	2.660	1.160	2.000	2.480	2.740
**Span**	2.077	1.811	1.837	1.660	1.586	2.139	2.130	2.047

Table S3. Particle sizes of prepared indomethacin suspension stabilized with 3% (m/v) polysorbate 80 dissolved in either citric buffer, pH 3.0 or acetate buffer, pH 4.0. Suspensions were stored over 28 days at 40 °C.

	**Citric buffer, pH 3.0**				**Acetate buffer, pH 4.0**			
	**Day 0**	**Day 7**	**Day 14**	**Day 28**	**Day 0**	**Day 7**	**Day 14**	**Day 28**
$\mathbf{D_{10}}$	0.346	0.421	0.407	0.457	0.335	0.385	0.421	0.434
$\mathbf{D_{50}}$	0.590	0.816	0.798	0.912	0.548	0.705	0.801	0.872
$\mathbf{D_{90}}$	1.490	1.790	1.910	2.010	1.080	1.590	1.690	1.900
**Span**	1.944	1.673	1.887	1.699	1.359	1.710	1.581	1.683

Table S4. Particle sizes of prepared indomethacin suspension stabilized with 3% (m/v) polysorbate 85 dissolved in either citric buffer, pH 3.0 or acetate buffer, pH 4.0. Suspensions were stored over 28 days at 40 °C.

	**Citric buffer, pH 3.0**				**Acetate buffer, pH 4.0**			
	**Day 0**	**Day 7**	**Day 14**	**Day 28**	**Day 0**	**Day 7**	**Day 14**	**Day 28**
$\mathbf{D_{10}}$	0.610	1.680	1.120	0.908	0.691	1.110	1.560	1.100
$\mathbf{D_{50}}$	1.440	3.530	2.970	2.620	1.690	2.920	3.310	2.650
$\mathbf{D_{90}}$	2.760	6.260	5.800	5.100	3.220	5.660	6.060	5.090
**Span**	1.489	1.298	1.575	1.600	1.498	1.557	1.358	1.509

Table S5. Particle sizes of prepared indomethacin suspension stabilized with 3% (m/v) poloxamer 188 dissolved in either citric buffer, pH 3.0 or acetate buffer, pH 4.0. Suspensions were stored over 28 days at 40 °C.

	**Citric buffer, pH 3.0**				**Acetate buffer, pH 4.0**			
	**Day 0**	**Day 7**	**Day 14**	**Day 28**	**Day 0**	**Day 7**	**Day 14**	**Day 28**
$\mathbf{D_{10}}$	0.351	0.375	0.394	0.379	0.328	0.344	0.345	0.377
$\mathbf{D_{50}}$	0.642	0.720	0.820	0.739	0.543	0.620	0.611	0.676
$\mathbf{D_{90}}$	1.910	1.890	2.130	1.960	1.360	1.700	1.590	1.640
**Span**	2.428	2.104	2.122	2.145	1.914	2.192	2.045	1.875

Table S6. Particle sizes of prepared indomethacin suspension stabilized with 3% (m/v) poloxamer 338 dissolved in either citric buffer, pH 3.0 or acetate buffer, pH 4.0. Suspensions were stored over 28 days at 40 °C.

	**Citric buffer, pH 3.0**				**Acetate buffer, pH 4.0**			
	**Day 0**	**Day 7**	**Day 14**	**Day 28**	**Day 0**	**Day 7**	**Day 14**	**Day 28**
**D_{10}**	0.363	0.386	0.451	0.392	0.343	0.400	0.390	0.397
**D_{50}**	0.722	0.848	1.040	0.857	0.648	0.877	0.786	0.793
**D_{90}**	2.360	2.390	2.800	2.380	2.190	2.370	2.140	2.150
**Span**	2.763	2.368	2.267	2.327	2.856	2.251	2.226	2.212

Table S7. Particle sizes of prepared indomethacin suspension stabilized with 3% (m/v) poloxamer 407 dissolved in either citric buffer, pH 3.0 or acetate buffer, pH 4.0. Suspensions were stored over 28 days at 40 °C.

	**Citric buffer, pH 3.0**				**Acetate buffer, pH 4.0**			
	**Day 0**	**Day 7**	**Day 14**	**Day 28**	**Day 0**	**Day 7**	**Day 14**	**Day 28**
**D_{10}**	0.367	0.375	0.463	0.383	0.357	0.406	0.407	0.395
**D_{50}**	0.705	0.752	0.994	0.778	0.667	0.898	0.808	0.792
**D_{90}**	2.380	2.090	2.670	2.120	2.230	2.350	2.010	2.090
**Span**	2.852	2.279	2.217	2.236	2.815	2.170	1.979	2.145

Table S8. Particle sizes of prepared indomethacin suspension stabilized with 3% (m/v) PVP K16-18 dissolved in either citric buffer, pH 3.0 or acetate buffer, pH 4.0. Suspensions were stored over 28 days at 40 °C.

	**Citric buffer, pH 3.0**				**Acetate buffer, pH 4.0**			
	**Day 0**	**Day 7**	**Day 14**	**Day 28**	**Day 0**	**Day 7**	**Day 14**	**Day 28**
**D_{10}**	0.346	0.490	0.533	0.491	0.362	0.370	0.387	0.376
**D_{50}**	0.665	1.180	1.290	1.220	0.643	0.739	0.766	0.690
**D_{90}**	2.710	2.490	2.800	2.530	2.990	2.500	2.680	2.330
**Span**	3.562	1.692	1.756	1.672	4.093	2.879	3.001	2.834

Table S9. Particle sizes of prepared indomethacin suspension stabilized with 3% (m/v) PVP K30 dissolved in either citric buffer, pH 3.0 or acetate buffer, pH 4.0. Suspensions were stored over 28 days at 40 °C.

	**Citric buffer, pH 3.0**				**Acetate buffer, pH 4.0**			
	**Day 0**	**Day 7**	**Day 14**	**Day 28**	**Day 0**	**Day 7**	**Day 14**	**Day 28**
**D_{10}**	0.337	0.354	0.371	0.362	0.309	0.325	0.335	0.334
**D_{50}**	0.619	0.711	0.735	0.692	0.529	0.572	0.576	0.564
**D_{90}**	2.510	2.090	2.320	2.110	2.060	1.970	1.720	1.600
**Span**	3.505	2.446	2.651	2.522	3.326	2.870	2.407	2.249

Table S10. Particle sizes of prepared indomethacin suspension stabilized with 3% (m/v) vitamin E TPGS dissolved in either citric buffer, pH 3.0 or acetate buffer, pH 4.0. Suspensions were stored over 28 days at 40 °C.

	Citric buffer, pH 3.0				Acetate buffer, pH 4.0			
	**Day 0**	**Day 7**	**Day 14**	**Day 28**	**Day 0**	**Day 7**	**Day 14**	**Day 28**
D_{10}	0.354	0.369	0.373	0.380	0.306	0.343	0.381	0.372
D_{50}	0.672	0.712	0.801	0.757	0.510	0.635	0.694	0.684
D_{90}	2.350	1.910	2.300	2.030	1.320	1.700	1.640	1.720
**Span**	2.968	2.162	2.407	2.180	1.977	2.141	1.807	1.964

Table S11. Particle sizes of prepared indomethacin suspension stabilized with 3% (m/v) SLS dissolved in either citric buffer, pH 3.0 or acetate buffer, pH 4.0. Suspensions were stored over 28 days at 40 °C.

	Citric buffer, pH 3.0				Acetate buffer, pH 4.0			
	**Day 0**	**Day 7**	**Day 14**	**Day 28**	**Day 0**	**Day 7**	**Day 14**	**Day 28**
D_{10}	0.367	0.417	0.442	0.411	0.364	0.432	0.424	0.421
D_{50}	0.674	0.869	0.926	0.858	0.654	0.906	0.873	0.868
D_{90}	1.810	2.090	2.000	2.040	1.660	2.060	1.970	1.980
**Span**	2.135	1.930	1.679	1.901	1.973	1.791	1.769	1.793

Table S12. Particle sizes of prepared naproxen suspension stabilized with 3% (m/v) polysorbate 20 dissolved in either citric buffer, pH 3.0 or acetate buffer, pH 4.0. Suspensions were stored over 28 days at 40 °C.

	**Citric buffer, pH 3.0**				**Acetate buffer, pH 4.0**			
	**Day 0**	**Day 7**	**Day 14**	**Day 28**	**Day 0**	**Day 7**	**Day 14**	**Day 28**
**D_{10}**	0.728	1.530	1.130	1.650	0.727	1.110	1.520	1.680
**D_{50}**	1.470	3.540	2.360	3.950	1.530	2.360	3.240	3.960
**D_{90}**	2.780	7.290	4.760	7.920	3.020	4.930	6.430	8.830
**Span**	1.396	1.625	1.542	1.596	1.503	1.619	1.516	1.807

Table S13. Particle sizes of prepared naproxen suspension stabilized with 3% (m/v) polysorbate 80 dissolved in either citric buffer, pH 3.0 or acetate buffer, pH 4.0. Suspensions were stored over 28 days at 40 °C.

	**Citric buffer, pH 3.0**				**Acetate buffer, pH 4.0**			
	**Day 0**	**Day 7**	**Day 14**	**Day 28**	**Day 0**	**Day 7**	**Day 14**	**Day 28**
**D_{10}**	0.505	0.993	0.874	1.200	0.593	0.752	1.040	1.130
**D_{50}**	1.020	2.040	1.810	2.400	1.190	1.580	2.160	2.300
**D_{90}**	2.570	3.970	3.800	4.560	2.770	3.800	4.170	4.480
**Span**	2.017	1.460	1.620	1.399	1.835	1.933	1.447	1.455

Table S14. Particle sizes of prepared naproxen suspension stabilized with 3% (m/v) polysorbate 85 dissolved in either citric buffer, pH 3.0 or acetate buffer, pH 4.0. Suspensions were stored over 28 days at 40 °C.

	**Citric buffer, pH 3.0**				**Acetate buffer, pH 4.0**			
	**Day 0**	**Day 7**	**Day 14**	**Day 28**	**Day 0**	**Day 7**	**Day 14**	**Day 28**
**D_{10}**	0.597	1.790	1.180	2.110	5.300	6.480	1.910	3.760
**D_{50}**	1.540	3.840	3.070	4.880	11.80	44.40	4.550	9.590
**D_{90}**	3.120	6.920	6.080	9.690	25.00	102.0	9.070	25.40
**Span**	1.644	1.333	1.599	1.549	1.681	2.365	1.573	2.260

Table S15. Particle sizes of prepared naproxen suspension stabilized with 3% (m/v) poloxamer 188 dissolved in either citric buffer, pH 3.0 or acetate buffer, pH 4.0. Suspensions were stored over 28 days at 40 °C.

	**Citric buffer, pH 3.0**				**Acetate buffer, pH 4.0**			
	**Day 0**	**Day 7**	**Day 14**	**Day 28**	**Day 0**	**Day 7**	**Day 14**	**Day 28**
**D_{10}**	0.843	1.310	1.260	1.380	0.915	1.190	1.100	1.090
**D_{50}**	1.820	2.880	3.390	3.230	2.190	3.220	2.710	3.010
**D_{90}**	3.890	5.880	57.80	6.860	16.10	42.40	6.560	12.20
**Span**	1.677	1.585	16.35	1.699	6.926	12.80	2.013	3.683

Table S16. Particle sizes of prepared naproxen suspension stabilized with 3% (m/v) poloxamer 338 dissolved in either citric buffer, pH 3.0 or acetate buffer, pH 4.0. Suspensions were stored over 28 days at 40 °C.

	**Citric buffer, pH 3.0**				**Acetate buffer, pH 4.0**			
	**Day 0**	**Day 7**	**Day 14**	**Day 28**	**Day 0**	**Day 7**	**Day 14**	**Day 28**
**D_{10}**	0.472	0.939	1.230	0.973	0.526	1.060	0.927	1.170
**D_{50}**	0.919	2.340	2.820	2.460	1.050	2.500	2.210	2.750
**D_{90}**	2.360	5.120	5.820	5.370	2.570	5.470	4.490	6.100
**Span**	2.052	1.788	1.630	1.786	1.955	1.761	1.837	1.790

Table S17. Particle sizes of prepared naproxen suspension stabilized with 3% (m/v) poloxamer 407 dissolved in either citric buffer, pH 3.0 or acetate buffer, pH 4.0. Suspensions were stored over 28 days at 40 °C.

	**Citric buffer, pH 3.0**				**Acetate buffer, pH 4.0**			
	**Day 0**	**Day 7**	**Day 14**	**Day 28**	**Day 0**	**Day 7**	**Day 14**	**Day 28**
**D_{10}**	0.496	0.990	1.240	1.190	0.550	1.280	1.350	1.170
**D_{50}**	1.060	2.640	3.010	3.100	1.140	2.950	3.150	2.960
**D_{90}**	2.650	6.040	6.500	6.860	2.780	6.410	6.610	6.600
**Span**	2.033	1.912	1.749	1.829	1.960	1.736	1.668	1.840

Table S18. Particle sizes of prepared naproxen suspension stabilized with 3% (m/v) PVP K16-18 dissolved in either citric buffer, pH 3.0 or acetate buffer, pH 4.0. Suspensions were stored over 28 days at 40 °C.

	**Citric buffer, pH 3.0**				**Acetate buffer, pH 4.0**			
	**Day 0**	**Day 7**	**Day 14**	**Day 28**	**Day 0**	**Day 7**	**Day 14**	**Day 28**
**D_{10}**	0.408	0.707	0.732	0.693	0.357	0.625	0.600	0.596
**D_{50}**	0.816	1.930	2.110	2.020	0.585	1.730	1.630	1.380
**D_{90}**	2.050	4.030	4.640	4.260	1.630	3.970	3.760	3.450
**Span**	2.014	1.720	1.855	1.770	2.172	1.932	1.944	2.069

Table S19. Particle sizes of prepared naproxen suspension stabilized with 3% (m/v) PVP K30 dissolved in either citric buffer, pH 3.0 or acetate buffer, pH 4.0. Suspensions were stored over 28 days at 40 °C.

	**Citric buffer, pH 3.0**				**Acetate buffer, pH 4.0**			
	**Day 0**	**Day 7**	**Day 14**	**Day 28**	**Day 0**	**Day 7**	**Day 14**	**Day 28**
**D_{10}**	0.365	0.488	0.624	0.624	0.366	0.405	0.412	0.404
**D_{50}**	0.637	1.270	1.880	1.880	0.612	0.806	0.760	0.721
**D_{90}**	2.170	3.200	4.20	4.340	1.820	2.490	2.190	2.070
**Span**	2.839	2.133	1.904	1.977	2.369	2.584	2.340	2.315

Table S20. Particle sizes of prepared naproxen suspension stabilized with 3% (m/v) vitamin E TPGS dissolved in either citric buffer, pH 3.0 or acetate buffer, pH 4.0. Suspensions were stored over 28 days at 40 °C.

	**Citric buffer, pH 3.0**				**Acetate buffer, pH 4.0**			
	**Day 0**	**Day 7**	**Day 14**	**Day 28**	**Day 0**	**Day 7**	**Day 14**	**Day 28**
**D_{10}**	0.524	0.851	0.747	0.833	0.430	0.604	0.739	0.661
**D_{50}**	1.100	1.80	1.710	1.790	0.817	1.310	1.650	1.430
**D_{90}**	2.580	3.840	4.310	3.930	2.470	4.210	4.520	4.060
**Span**	1.873	1.662	2.081	1.729	2.496	2.762	2.294	2.380

Table S21. Particle sizes of prepared naproxen suspension stabilized with 3% (m/v) SLS dissolved in either citric buffer, pH 3.0 or acetate buffer, pH 4.0. Suspensions were stored over 28 days at 40 °C.

	**Citric buffer, pH 3.0**				**Acetate buffer, pH 4.0**			
	**Day 0**	**Day 7**	**Day 14**	**Day 28**	**Day 0**	**Day 7**	**Day 14**	**Day 28**
**D_{10}**	0.935	1.900	1.690	1.720	1.350	1.400	1.820	1.780
**D_{50}**	3.860	4.650	4.850	4.120	3.570	4.790	5.340	4.770
**D_{90}**	11.70	10.80	14.10	8.810	9.510	17.10	24.00	13.90
**Span**	2.768	1.905	2.556	1.722	2.287	3.271	4.121	2.545

Table S22. Particle sizes of dual suspensions containing both indomethacin and naproxen stabilized with 3% (m/v) polysorbate 20 dissolved in citric buffer, pH 3.0. Suspensions were stored over 28 days at 40 °C. The particle sizes were analyzed with Mie-Theory or Fraunhofer Approximation.

	**Mie-Theory**				**Fraunhofer approximation**			
	**Day 0**	**Day 7**	**Day 14**	**Day 28**	**Day 0**	**Day 7**	**Day 14**	**Day 28**
**D_{10}**	0.369	0.469	0.801	0.657	0.405	0.518	0.602	0.637
**D_{50}**	0.632	1.020	2.310	1.720	0.851	1.140	2.120	1.650
**D_{90}**	1.520	2.540	4.610	3.790	1.650	2.450	4.530	3.670
**Span**	1.829	2.025	1.652	1.820	1.460	1.700	1.855	1.843

Table S23. Particle sizes of dual suspensions containing both indomethacin and naproxen stabilized with 3% (m/v) polysorbate 80 dissolved in citric buffer, pH 3.0. Suspensions were stored over 28 days at 40 °C. The particle sizes were analyzed with Mie-Theory or Fraunhofer Approximation.

	**Mie-Theory**				**Fraunhofer approximation**			
	**Day 0**	**Day 7**	**Day 14**	**Day 28**	**Day 0**	**Day 7**	**Day 14**	**Day 28**
**D_{10}**	0.356	0.421	0.445	0.451	0.367	0.478	0.531	0.540
**D_{50}**	0.578	0.847	0.912	0.910	0.781	1.030	1.070	1.070
**D_{90}**	1.340	2.080	2.040	1.960	1.510	2.070	2.040	1.990
**Span**	1.702	1.956	1.747	1.661	1.463	1.544	1.415	1.357

Table S24. Particle sizes of dual suspensions containing both indomethacin and naproxen stabilized with 3% (m/v) polysorbate 85 dissolved in citric buffer, pH 3.0. Suspensions were stored over 28 days at 40 °C. The particle sizes were analyzed with Mie-Theory or Fraunhofer Approximation.

	**Mie-Theory**				**Fraunhofer approximation**			
	**Day 0**	**Day 7**	**Day 14**	**Day 28**	**Day 0**	**Day 7**	**Day 14**	**Day 28**
**D_{10}**	0.801	1.540	1.590	1.300	0.572	1.070	1.180	0.937
**D_{50}**	2.290	3.490	3.270	2.950	2.200	3.410	3.240	2.840
**D_{90}**	4.160	6.540	5.710	5.340	4.160	6.480	5.730	5.330
**Span**	1.467	1.430	1.261	1.371	1.631	1.585	1.408	1.545

Table S25. Particle sizes of dual suspensions containing both indomethacin and naproxen stabilized with 3% (m/v) poloxamer 188 dissolved in citric buffer, pH 3.0. Suspensions were stored over 28 days at 40 °C. The particle sizes were analyzed with Mie-Theory or Fraunhofer Approximation.

	**Mie-Theory**				**Fraunhofer approximation**			
	**Day 0**	**Day 7**	**Day 14**	**Day 28**	**Day 0**	**Day 7**	**Day 14**	**Day 28**
**D_{10}**	0.357	0.409	0.407	0.421	0.367	0.466	0.445	0.470
**D_{50}**	0.596	0.896	0.750	0.836	0.825	1.090	0.951	1.020
**D_{90}**	1.590	2.650	1.800	2.290	1.690	2.560	1.860	2.230
**Span**	2.070	2.496	1.863	2.238	1.601	1.925	1.490	1.727

Table S26. Particle sizes of dual suspensions containing both indomethacin and naproxen stabilized with 3% (m/v) poloxamer 338 dissolved in citric buffer, pH 3.0. Suspensions were stored over 28 days at 40 °C. The particle sizes were analyzed with Mie-Theory or Fraunhofer Approximation.

	**Mie-Theory**				**Fraunhofer approximation**			
	**Day 0**	**Day 7**	**Day 14**	**Day 28**	**Day 0**	**Day 7**	**Day 14**	**Day 28**
**D_{10}**	0.374	0.524	0.420	0.435	0.420	0.534	0.484	0.509
**D_{50}**	0.722	1.300	0.874	0.927	0.998	1.330	1.060	1.090
**D_{90}**	2.370	3.710	2.370	2.410	2.320	3.420	2.300	2.330
**Span**	2.775	2.450	2.224	2.126	1.900	2.173	1.707	1.673

Table S27. Particle sizes of dual suspensions containing both indomethacin and naproxen stabilized with 3% (m/v) poloxamer 407 dissolved in citric buffer, pH 3.0. Suspensions were stored over 28 days at 40 °C. The particle sizes were analyzed with Mie-Theory or Fraunhofer Approximation.

	**Mie-Theory**				**Fraunhofer approximation**			
	**Day 0**	**Day 7**	**Day 14**	**Day 28**	**Day 0**	**Day 7**	**Day 14**	**Day 28**
**D_{10}**	0.363	0.454	0.404	0.420	0.392	0.503	0.452	0.484
**D_{50}**	0.650	1.180	0.765	0.843	0.917	1.270	0.972	1.030
**D_{90}**	2.080	3.590	2.030	2.280	2.070	3.430	2.020	2.220
**Span**	2.643	2.645	2.120	2.204	1.835	2.292	1.611	1.686

Table S28. Particle sizes of dual suspensions containing both indomethacin and naproxen stabilized with 3% (m/v) PVP K16-K18 dissolved in citric buffer, pH 3.0. Suspensions were stored over 28 days at 40 °C. The particle sizes were analyzed with Mie-Theory or Fraunhofer Approximation.

	**Mie-Theory**				**Fraunhofer approximation**			
	**Day 0**	**Day 7**	**Day 14**	**Day 28**	**Day 0**	**Day 7**	**Day 14**	**Day 28**
D_{10}	0.399	0.564	0.451	0.485	0.533	0.642	0.621	0.627
D_{50}	0.803	1.310	1.050	1.170	1.090	1.380	1.240	1.290
D_{90}	2.380	2.930	2.390	2.670	2.200	2.750	2.290	2.550
**Span**	2.473	1.807	1.834	1.869	1.530	1.529	1.352	1.490

Table S29. Particle sizes of dual suspensions containing both indomethacin and naproxen stabilized with 3% (m/v) PVP K30 dissolved in citric buffer, pH 3.0. Suspensions were stored over 28 days at 40 °C. The particle sizes were analyzed with Mie-Theory or Fraunhofer Approximation.

	**Mie-Theory**				**Fraunhofer approximation**			
	**Day 0**	**Day 7**	**Day 14**	**Day 28**	**Day 0**	**Day 7**	**Day 14**	**Day 28**
D_{10}	0.325	0.381	0.368	0.386	0.359	0.477	0.458	0.501
D_{50}	0.565	0.775	0.681	0.784	0.908	1.070	0.979	1.060
D_{90}	2.110	2.440	1.960	2.210	2.100	2.330	1.960	2.150
**Span**	3.161	2.656	2.340	2.323	1.913	1.729	1.533	1.555

Table S30. Particle sizes of dual suspensions containing both indomethacin and naproxen stabilized with 3% (m/v) vitamin E TPGS dissolved in citric buffer, pH 3.0. Suspensions were stored over 28 days at 40 °C. The particle sizes were analyzed with Mie-Theory or Fraunhofer Approximation.

	**Mie-Theory**				**Fraunhofer approximation**			
	**Day 0**	**Day 7**	**Day 14**	**Day 28**	**Day 0**	**Day 7**	**Day 14**	**Day 28**
$\mathbf{D_{10}}$	0.348	0.416	0.429	0.430	0.368	0.483	0.501	0.508
$\mathbf{D_{50}}$	0.592	0.914	0.845	0.878	0.843	1.090	1.020	1.040
$\mathbf{D_{90}}$	1.750	2.440	1.980	2.160	1.820	2.380	1.980	2.130
**Span**	2.375	2.217	1.830	1.966	1.718	1.741	1.450	1.557

Table S31. Particle sizes of dual suspensions containing both indomethacin and naproxen stabilized with 3% (m/v) SLS dissolved in citric buffer, pH 3.0. Suspensions were stored over 28 days at 40 °C. The particle sizes were analyzed with Mie-Theory or Fraunhofer Approximation.

	**Mie-Theory**				**Fraunhofer approximation**			
	**Day 0**	**Day 7**	**Day 14**	**Day 28**	**Day 0**	**Day 7**	**Day 14**	**Day 28**
$\mathbf{D_{10}}$	0.389	0.502	0.476	0.636	0.430	0.530	0.531	0.595
$\mathbf{D_{50}}$	0.695	1.120	1.030	1.870	0.899	1.190	1.140	1.590
$\mathbf{D_{90}}$	1.790	3.480	2.820	8.630	1.820	3.170	2.610	7.720
**Span**	2.020	2.611	2.275	4.374	1.547	2.191	1.819	4.504

Table S32. Particle sizes of dual suspensions containing both indomethacin and naproxen stabilized with 3% (m/v) vitamin E TPGS and polysorbate 85 dissolved in citric buffer, pH 3.0. Suspensions were stored over 28 days at 40 °C. The particle sizes were analyzed with Mie-Theory.

	**Day 0**	**Day 7**	**Day 14**	**Day 28**
**D_{10}**	0.350	0.419	0.431	0.430
**D_{50}**	0.640	0.879	0.947	0.896
**D_{90}**	1.910	2.330	2.460	2.170
**Span**	2.543	2.174	2.143	1.937

Table S33. Particle sizes of dual suspensions containing both indomethacin and naproxen stabilized with 3% (m/v) poloxamer 188 and PVP K30 dissolved in citric buffer, pH 3.0. Suspensions were stored over 28 days at 40 °C. The particle sizes were analyzed with Mie-Theory.

	**Day 0**	**Day 7**	**Day 14**	**Day 28**
**D_{10}**	0.347	0.426	0.449	0.451
**D_{50}**	0.631	0.976	1.030	1.010
**D_{90}**	1.830	2.470	2.400	2.370
**Span**	2.354	2.088	1.893	1.894

Table S34. Particle sizes of dual suspensions containing both indomethacin and naproxen stabilized with 3% (m/v) polysorbate 85 and poloxamer 338 dissolved in citric buffer, pH 3.0. Suspensions were stored over 28 days at 40 °C. The particle sizes were analyzed with Mie-Theory.

	**Day 0**	**Day 7**	**Day 14**	**Day 28**
**D_{10}**	0.341	0.388	0.381	0.389
**D_{50}**	0.611	0.795	0.755	0.785
**D_{90}**	1.910	2.380	2.260	2.350
**Span**	2.565	2.492	2.481	2.490

Table S35. Particle sizes of dual suspensions containing both indomethacin and naproxen stabilized with 3% (m/v) vitamin E TPGS and PVP K16-18 dissolved in citric buffer, pH 3.0. Suspensions were stored over 28 days at 40 °C. The particle sizes were analyzed with Mie-Theory.

	**Day 0**	**Day 7**	**Day 14**	**Day 28**
**D_{10}**	0.329	0.405	0.430	0.415
**D_{50}**	0.563	0.825	0.983	0.859
**D_{90}**	1.680	2.100	2.500	2.110
**Span**	2.390	2.054	2.105	1.974

Table S36. Particle sizes of dual suspensions containing both indomethacin and naproxen stabilized with 3% (m/v) polysorbate 20 and PVP K16-18 dissolved in citric buffer, pH 3.0. Suspensions were stored over 28 days at 40 °C. The particle sizes were analyzed with Mie-Theory.

	**Day 0**	**Day 7**	**Day 14**	**Day 28**
**D_{10}**	0.367	0.469	0.587	0.609
**D_{50}**	0.663	1.060	1.450	1.430
**D_{90}**	1.690	2.530	3.280	3.190
**Span**	2.002	1.944	1.859	1.807

Figures

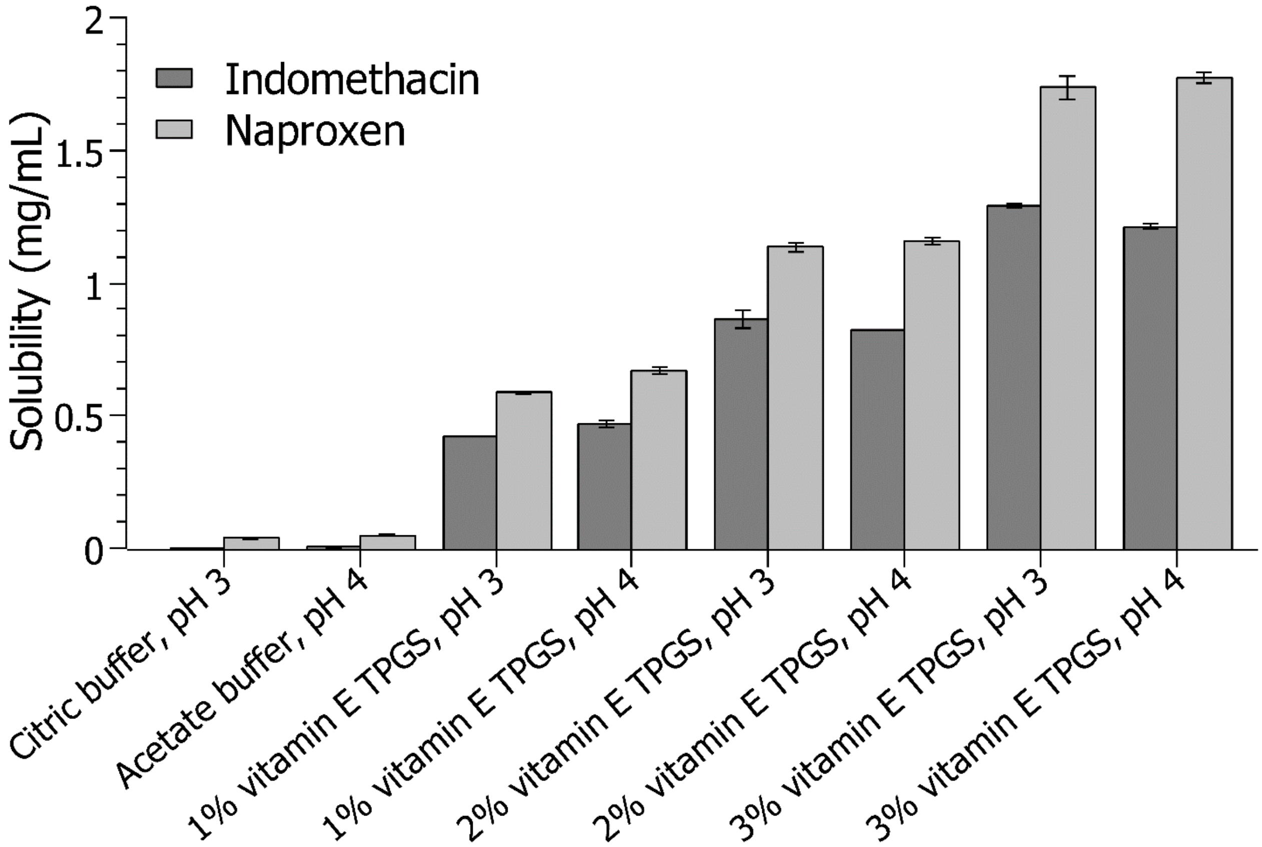

Fig. S1. Overview of the solubility of indomethacin and naproxen in different buffer systems and increasing concentration of vitamin E TPGS prepared with either citric buffer, pH 3.0 or acetate buffer, pH 4.0 (n = 3).

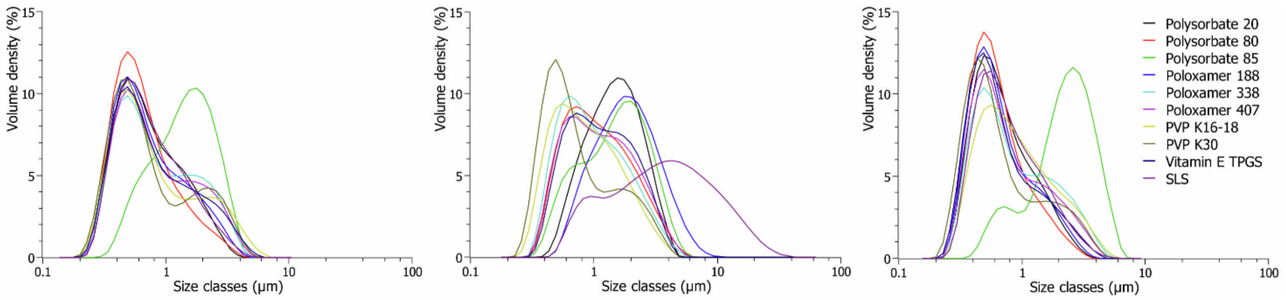

Fig. S2. Overview of obtained particle size distributions right after milling from the stabilizer screening of indomethacin suspensions (left), naproxen suspensions (middle), and dual suspensions containing both drug compounds analyzed with Mie-theory.
